# The ‘prostate-muscle index’: a simple pelvic cavity measurement predicting estimated blood loss and console time in robot-assisted radical prostatectomy

**DOI:** 10.1038/s41598-022-16202-6

**Published:** 2022-07-13

**Authors:** Naoki Kimura, Yuta Yamada, Yuta Takeshima, Masafumi Otsuka, Nobuhiko Akamatsu, Yuji Hakozaki, Jimpei Miyakawa, Yusuke Sato, Yoshiyuki Akiyama, Daisuke Yamada, Tetsuya Fujimura, Haruki Kume

**Affiliations:** 1 Department of Urology, National Center for Global Health and Medicine, Shinjuku-Ku, Tokyo, Japan; 2 Department of Urology, Graduate School of Medicine, The University of Tokyo, 7-3-1, Hongo, Bunkyo-Ku, Tokyo, 113-8655 Japan; 3 Division of Innovative Cancer Therapy, The Advanced Clinical Research Center, The Institute of Medical Science, The University of Tokyo, Minato-Ku, Tokyo, Japan; 4 Aoi-Clinic, Yokohama City, Kanagawa Japan; 5 Department of Radiology, Nerima Hikarigaoka Hospital, Nerima-Ku, Tokyo, Japan; 6 Department of Urology, Jichi Medical University, Shimotsuke City, Tochigi Japan

**Keywords:** Oncology, Urology

## Abstract

This study was to show the impact of ‘prostate-muscle index (PMI)’, which we developed as a novel pelvic cavity measurement, in patients undergoing robot-assisted radical prostatectomy (RARP). We defined PMI as the ‘distance between the inner edge of the obturator internus muscle and the lateral edge of the prostate at the magnetic resonance imaging (MRI) slice showing the maximum width of the prostate’. Seven hundred sixty patients underwent RARP at the University of Tokyo Hospital from November 2011 to December 2018. MRI results were unavailable in 111 patients. In total, 649 patients were eligible for this study. Median values of blood loss and console time were 300 mL and 168 min. In multivariate analysis, body mass index (BMI), prostate volume-to-pelvic cavity index (PV-to-PCI), PMI, and surgical experience were significantly associated with blood loss > 300 mL (*P* = 0.0002, 0.002, < 0.0001, and 0.006 respectively). Additionally, BMI, PMI, and surgical experience were also significantly associated with console time > 160 min in multivariate analysis (*P* = 0.04, 0.004, and < 0.0001, respectively). In conclusion, PMI may provide useful information to surgeons and patients in preoperative decision-making.

## Introduction

Radical prostatectomy (RP) is the gold-standard treatment for clinically localized prostate cancer. The surgical method has shifted towards robotic surgery regarding RP since robot-assisted radical prostatectomy (RARP) was first introduced in 2001^1^. Robotic equipment has provided advantages such as the 3-dimensional magnified view of the prostatic and pelvic anatomy^2,3^ and multi-junctional forceps that allow for excellent flexibility and maneuverability^4^. In addition, RARP is carried out under intra-peritoneal pressure that decreases massive bleeding by CO_2_ insufflation^5^. Yet despite these technical advances, cases with complications such as excessive blood loss remain^6^, and there exists a further need to identify risk factors associated with surgical outcomes.

**Figure 1:**
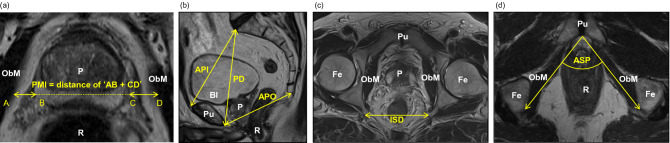
Definition of parameters in MRI measurements. (**a**) Axial section of MRI on T2 showing the maximum area of the prostate. A dashed-yellow line ‘BC’ indicates the maximum length of the width of the prostate at the section showing the maximum area of the prostate. The line is extended where it crosses the lateral edge of the obturator internus muscle. Point A and D were defined as the point crossing the lateral edge of the obturator internus muscle. ‘Prostate-muscle index (PMI)’ was defined as the sum of distance 'AB + CD' shown in a yellow line with arrowheads. (**b**) Sagittal section of MRI on T2 showing the pelvic cavity. ‘Anteroposterior diameter of the pelvic inlet (API)’, ‘pelvic depth (PD)’, and ‘anteroposterior diameter of the pelvic outlet (APO)’ were defined as the ‘diameter from the sacral promontory to the most superior aspect of the pubic symphysis’ (mm), ‘diameter from the sacral promontory to the most inferior point of the pubic symphysis’ (mm), ‘diameter from the inferior aspect of the pubic symphysis to the tip of the coccyx’ (mm). (**c**, **d**) Axial section of MRI onT2 showing the measurement of ISD and ASP. ‘Interspinous distance (ISD)’ was defined as ‘the narrowest distance between tips of the ischial spines’ (mm). ‘Angle of the symphysis pubis (ASP)’ was also measured. P: prostate, Fe: femoral bone, Pu: pubic bone, R: rectum, ObM: obturator internus muscle, Bl: bladder.

In the present study, we focused on preoperative pelvic magnetic resonance imaging (MRI) to evaluate risk factors of surgical performance. Although some reports show that ‘prostate volume-to-pelvic cavity index (PV-to-PCI)’ correlates positively with the amount of blood loss^7,8^, the calculation is rather complex since it requires 6 parameters. Hence, we developed a novel predictor which we termed ‘prostate-muscle index (PMI)’. PMI is defined as ‘the distance between the inner edge of the obturator internus muscle and the lateral edge of the prostate at the MRI section showing the maximum width of the prostate’ (Fig. 1a) and it requires only 1 parameter. In this study, we investigated the relationship between this novel predictor and perioperative morbidity.

## Methods

### Patients’ characteristics and surgical techniques

Seven hundred and sixty patients underwent RARP for prostate cancer at the University of Tokyo Hospital from November 2011 to December 2018. One hundred eleven patients were excluded from this analysis since preoperative pelvic MRI was not performed in these patients. A total of 649 patients were analyzed for clinicopathological variables, surgery time, console time, and blood loss. Clinicopathological variables included age, BMI, preoperative prostate-specific antigen (PSA) level, PV, pathological T stage, Gleason score (GS), and surgical margin. Additionally, we analyzed surgery-related parameters, including nerve-sparing and surgical experience.

All patients underwent RARP using the da Vinci surgical robot system (Intuitive Surgical Incorporation, Sunnyvale, CA) and RARP procedures were carried out by transperitoneal approach with 6-port technique, as described in our previous studies^9^. All surgeries were performed at the pneumoperitoneum pressure of 10 mmHg except for procedure regarding the management of dorsal vein complex, which was carried out at the pressure of 15 mmHg.

This study was accepted by the ‘Ethics Committee of the Tokyo University Hospital’ (# 3124), and was performed in accordance with the Helsinki declaration. We obtained written informed consent from each patient before surgery.

### MRI measurements and assessment

Pelvic MRI was performed using a 3-T whole-body MRI system (Signa HDx; GE Healthcare, Milwaukee, Wis) by using a 12-channel phased-array head coil. Methods of measuring MRI-related parameters including PMI were supervised by a radiologist (NA). All measurements were defined by two urologists (NK and MO). When the measurement values were different, YY supervised and determined the measurement value.

In this study, we used 4 known MRI-measurement parameters, namely PV, PCI, ASP, and APO. In addition, we proposed a novel parameter ‘PMI’, and compared its clinical performance and significance in the RARP procedure. PV was calculated by the ellipsoid formula, PV = width (cm) × length (cm) × height (cm) × (π/6)^10^. PCI was calculated by the following calculation formula, PCI = ‘anteroposterior diameter of the pelvic inlet’ (API: diameter from the sacral promontory to the most superior aspect of the pubic symphysis) (mm) × ‘interspinous distance’ (ISD) (mm) / ‘pelvic depth’ (PD: diameter from the sacral promontory to the most inferior point of the pubic symphysis) (mm) (Fig. 1b, c)^7,11^. ASP was measured as ‘the angle of the symphysis pubis’ (Fig. 1d) and APO was ‘diameter from the inferior aspect of the pubic symphysis to the tip of the coccyx’, respectively (Fig. 1b). PMI measurement was performed on the axial T2 weighted image section that showed the maximum width of the prostate. As shown in Fig. 1a, line BC shows the maximum length of the width of the prostate at the section showing the maximum area of the prostate and this line is extended to the point A and D, where the line BC crosses the lateral edge of the obturator internus muscle. The distance between AB and CD was measured and PMI was defined as the sum of distance 'AB + CD'. Therefore, PMI can be defined as ‘the distance between the inner edge of the obturator internus muscle and the lateral edge of the prostate at the MRI section showing the maximum width of the prostate’.

### Statistical analyses

We used the JMP 15.0 software (SAS Institute, Cary, NC, USA) for statistical analysis. The median or quartile value of all parameters were rounded to the integer value and were used as cut-off values. The correlation among blood loss, console time, BMI, PV, PCI, ASP, APO, prostate width, prostate length, and prostate height and PMI were evaluated by Wilcoxon rank sum test. Multivariate logistic regression modeling was used to examine the association between increased blood loss and each clinical parameter or prolonged console time. The width, length, and height of the prostate, PV-to-PCI, and PV were all prostate-related measurements and therefore PV-to-PCI, the strongest factor, was included in the multivariate analysis. We also carried out a statistical analysis regarding the predictors of blood loss and console time in a subset of patients undergoing RARP with or without pelvic lymphadenectomy. The cutoff values in these subsets of patients were determined by the same method as the entire cohort. Statistically significant P-value was defined as less than 0.05.

## Results

### Low PMI is associated with more blood loss and longer console time

Median values (IQR) of body mass index (BMI), prostate volume (PV), console time, and blood loss were 23.9 (21.9–25.6), 28.6 cm^3^ (22.8–37.7), 168 min (129–208), and 300 mL (100–500), respectively (Supplementary Table S1). The nerve-sparing procedure was performed in 171 patients (unilateral 161, bilateral 10) and 57 patients (8.8%) had positive surgical margins in pT2 patients. The median values of ‘angle of the symphysis pubis (ASP)’, ‘anteroposterior diameter of the pelvic outlet (APO)’, PMI, and PV-to-PCI were 72.8 degrees (68.8–77.0), 81.3 mm (75.4–86.8), 7.9 mm (4.4–11.6), and 3.6 (2.8–4.8), respectively (Supplementary Table S2). When the cutoff value of PMI was set to the lower quartile value (4.4 mm) and rounded to a clinically useful value (5.0 mm), shorter PMI was associated with more blood loss, longer console time, higher BMI, larger PV, lower PCI, higher PV-to-PCI, and longer width, length, and height of the prostate (all values *P* < 0.0001). Additionally, PMI was significantly associated with lower APO and ASP (*P* = 0.03 and 0.03, respectively). Risk of blood transfusion tend to be higher in low PMI group (≤ 5 mm) (Table 1).

**Table 1:** The relationship between PMI and other parameters (N = 649).

	PMI ≤ 5.0 (N = 181)	PMI > 5.0 (N = 468)	*P*-value
Median blood loss, mL, (IQR)	600 (400–852)	200 (100–350)	** < 0.0001**
Median console time, min, (IQR)	186 (155–223)	156 (123–202)	** < 0.0001**
Median BMI, kg/m^2^, (IQR)	24.8 (23.1–26.2)	23.5 (21.5–25.1)	** < 0.0001**
Median PV, cm^3^, (IQR)	34.4 (27.7–44.0)	27.2 (21.4–35.4)	** < 0.0001**
Median PCI, (IQR)	7.5 (7.1–8.0)	7.8 (7.4–8.4)	** < 0.0001**
Median ASP, degree, (IQR)	71.6 (67.7–76.2)	73.2 (69.1–77.4)	**0.03**
Median APO, mm, (IQR)	82.3 (76.8–88.0)	81.1 (74.8–86.4)	**0.03**
Median PV-to-PCI, (IQR)	4.6 (3.5–5.7)	3.3 (2.7–4.3)	** < 0.0001**
Median prostate width, mm, (IQR)	50.3 (46.7–54.0)	47.0 (43.7–50.9)	** < 0.0001**
Median prostate length, mm, (IQR)	38.9 (34.4–43.0)	35.1 (31.2–38.7)	** < 0.0001**
Median prostate height, mm, (IQR)	35.4 (30.5–38.7)	32.0 (28.8–35.9)	** < 0.0001**
Blood transfusion rate, cases, (%)	13 (7.1)	17 (3.6)	0.053

PMI > 5.0 mm was defined as ‘high PMI’. Statistical analyses were performed by the Wilcoxon rank-sum test and the chi-square test. *PMI* Prostate-muscle index, *BMI* Body mass index, *PV* Prostate volume, *PCI* Pelvic cavity index, *ASP* Angle of the symphysis pubis, *APO* Anteroposterior diameter of the pelvic outlet, *IQR* Interquartile range.

### PMI is an independent risk factor of estimated blood loss and console time in RARP

BMI, PV, PV-to-PCI, PMI, surgical experience, and the measurements of the prostate were significantly associated with ‘blood loss > 300 mL’ in the univariate analysis. In the multivariate analysis, BMI, PV-to-PCI, PMI, and surgical experience remained significant predictors of ‘blood loss > 300 mL’ (OR: 2.04 [1.39–3.00], 1.82 [1.23–2.70], 7.79 [4.72–12.8], 1.69 [1.16–2.47], respectively; Table 2). In addition, BMI, PV, PV-to-PCI, PMI, surgical experience, and the width/height of the prostate were significant predictors of ‘console time > 160 min’ in the univariate analysis. In the multivariate analysis, BMI, PMI, and surgical experience remained significant predictors of ‘console time > 160 min’ (OR: 1.63 [1.00–2.67], 2.28 [1.29–4.04], 16.1 [9.92–26.1], respectively; Table 3). Concerning ‘surgical margin’ and ‘urinary continence’, there were no significant associations with PMI (Supplementary Table S3).

**Table 2:** Univariate and multivariate analyses of factors associated with ‘blood loss > 300 mL’ in the entire cohort including patients undergoing RARP with/without pelvic lymphadenectomy (N = 649).

Parameters	Univariate		Multivariate	
	OR (95% CI)	P-value	OR (95% CI)	*P*-value
BMI (kg/m^2^) (BMI ≥ 24.0 vs. < 24.0)	2.57 (1.87–3.54)	** < 0.0001**	2.04 (1.39–3.00)	**0.0002**
PV (cm^3^) (PV ≥ 30.0 vs. < 30.0)	2.26 (1.64–3.11)	** < 0.0001**		
PV-to-PCI (PV-to-PCI ≥ 4.0 vs. < 4.0)	2.88 (2.04–4.06)	** < 0.0001**	1.82 (1.23–2.70)	**0.002**
PMI (mm) (PMI ≤ 5.0 vs. > 5.0)	9.72 (6.20–15.2)	** < 0.0001**	7.79 (4.72–12.8)	** < 0.0001**
Surgical experience (Volume ≤ 25 vs. > 25)	1.94 (1.42–2.66)	** < 0.0001**	1.69 (1.16–2.47)	**0.006**
ASP (degree) (ASP < 73.0 versus ≥ 73.0)	1.21 (0.88–1.64)	0.2		
APO (mm) (APO < 81.0 vs. ≥ 81.0)	0.73 (0.52–1.01)	0.06		
Prostate width (mm) (> 50.0 vs. ≤ 50.0)	1.61 (1.16–2.24)	**0.0040**		
Prostate length (mm) (≥ 30.0 vs. < 30.0)	1.98 (1.22–3.21)	**0.006**		
Prostate height (mm) (≥ 35.0 vs. < 35.0)	1.96 (1.41–2.71)	** < 0.0001**		
Lymph node dissection	1.41 (0.97–2.07)	0.08		

Logistic regression models were used for univariate and multivariate analyses. *P*-value of < 0.05 was considered to be statistically significant. *OR* Odds ratio, *CI* Confidence interval, *BMI* Body mass index, *PV* Prostate volume, *PCI* Pelvic cavity index, *PMI* Prostate-muscle index, *ASP* Angle of the symphysis pubis, *APO* Anteroposterior diameter of the pelvic outlet.

**Table 3:** Univariate and multivariate analyses of factors associated with ‘console time > 160 min’ in PC patients in the entire cohort including patients undergoing RARP with/without pelvic lymphadenectomy (N = 649).

Parameters	Univariate		Multivariate	
	OR (95% CI)	P-value	OR (95% CI)	*P*-value
BMI (kg/m^2^) (BMI ≥ 24.0 vs. < 24.0)	1.61 (1.13–2.30)	**0.009**	1.63 (1.00–2.67)	**0.04**
PV (cm^3^) (PV ≥ 30.0 vs. < 30.0)	1.52 (1.07–2.18)	**0.02**		
PV-to-PCI (PV-to-PCI ≥ 4.0 vs. < 4.0)	1.79 (1.22–2.62)	**0.003**	1.43 (0.86–2.380)	0.1
PMI (mm) (PMI ≤ 5.0 vs. > 5.0)	2.88 (1.90–4.35)	** < 0.0001**	2.28 (1.29–4.04)	**0.004**
Surgical experience (Volume ≤ 25 vs. > 25)	14.8 (9.63–22.7)	** < 0.0001**	16.1 (9.92–26.1)	** < 0.0001**
ASP (degree) (ASP < 73.0 vs. ≥ 73.0)	1.41 (0.99–2.00)	0.051		
APO (mm) (APO < 81.0 vs. ≥ 81.0)	0.85 (0.58–1.25)	0.4		
Prostate width (mm) (> 50.0 vs. ≤ 50.0)	1.56 (1.08–2.25)	**0.02**		
Prostate length (mm) (≥ 30.0 vs. < 30.0)	1.09 (0.74–1.61)	0.6		
Prostate height (mm) (≥ 35.0 vs. < 35.0)	1.50 (1.05–2.10)	**0.02**		

Logistic regression models were used for univariate and multivariate analyses. *P*-value of < 0.05 was considered to be statistically significant. *OR* Odds ratio, *CI* Confidence interval, *BMI* Body mass index, *PV* Prostate volume, *PCI* Pelvic cavity index, *PMI* Prostate-muscle index, *ASP* Angle of the symphysis pubis, *APO* Anteroposterior diameter of the pelvic outlet.

### PMI was a significant predictor of blood loss and console time in a subset of patients undergoing RARP without pelvic lymphadenectomy

In a subset of patients undergoing RARP without pelvic lymphadenectomy, multivariate analysis showed that PMI, BMI, and PV-to-PCI were significant factors of blood loss and PMI, BMI, and surgical experience were significant factors associated with console time (Supplementary Tables S4, S5). To note, PMI showed an OR of 9.96 [5.45–18.2] in association with blood loss and an OR of 2.28 [1.29–4.04] in association with console time (Supplementary Tables S4, S5).

### PMI was a significant predictor of blood loss in a subset of patients undergoing RARP with pelvic lymphadenectomy

In a subset of patients with pelvic lymphadenectomy, multivariate analysis showed that PMI and surgical experience were significant factors of blood loss (OR: 3.60 [1.43–9.00] and 2.69 [1.19–6.05], respectively; Supplementary Table S6). On the contrary, PMI did not show statistical significance on association with longer console time in this subset of patients (Supplementary Table S7). To note, surgical experience was the only significant factor associated with longer console time in this spectrum of patients (Supplementary Table S7). Transfusion rate was significantly higher in low PMI group ().

## Discussion

Various parameters of pelvic measurements are associated with RARP outcomes including blood loss and console time. However, many of these parameters are composed of skeletal measurements alone^11,12^, although the pelvic cavity is composed of not only the pelvic bone structure but also the pelvic muscles, fat, and other soft tissue that lie above the bony structure. The musculature is highly important when discussing outcomes of RP, as reflected in studies where preservation of the levator ani muscle is reported to benefit early urinary continence recovery after RP^13^. Accordingly, measurements reflecting the size of the pelvic cavity should contain elements regarding this muscle in addition to the bony structure. However, the clinical significance of pelvic musculature in terms of surgical performance in the RARP procedure has not been reported. Anatomically, the obturator internus muscle is located at the bottom of the lateral pelvic walls directly adjacent to the prostate, and it is one of the key components that construct the pelvic cavity^14,15^. Therefore, we sought to identify PMI as a novel pelvic parameter that reflects the influence of the pelvic muscle.

PMI may be a pelvic cavity marker that reflects the influence of BMI. In the present study, PMI was inversely correlated with BMI. Skeletal muscle volume is higher in high BMI patients than in low or normal BMI patients^16^. Furthermore, the fat tends to accumulate in muscles in patients with high BMI^17^. Taken together, muscle volume of the obturator internus muscle may increase with the increment of BMI level, and may conversely decrease the size of the pelvic cavity. It is of interest that despite this correlation, both BMI and PMI remained independent predictors of blood loss in multivariate analysis.

It has been reported that the RARP procedure significantly reduced the risk of blood loss compared with open RP^18^. Although excessive bleeding occasionally occurs during the management of the dorsal vein complex (DVC) and vascular pedicle in the RARP procedure^19^, any possible site may be the source of a vast bleeding when dissecting the prostate. A smaller pelvic cavity restricts the range of robotic arm motion and may lead to difficulty in managing excessive bleeding. Although a previous study failed to show an association between the pelvic cavity and surgical performance in RARP procedure^20^, this study did not consider the impact of PV. In theory, the surgical performance may be influenced by both pelvic cavity and PV since the actual cavity is determined by both elements. Indeed, PV-to-PCI, which is a parameter containing both elements of the pelvic cavity and PV, were significantly associated with excessive bleeding and console time^7,8^. However, PV-to-PCI is not practical in clinical use since it requires as many as 6 parameters. Conversely, PMI uses only a single parameter, and urologists may calculate this parameter easily.

The clinical significance may potentially be influenced by the pneumoperitoneum pressure. In theory, higher intraperitoneal pressure may provide more working space but may as well be associated with more complications^21^. A study by Rohloff et al. showed that length of stay and postoperative ileus rates were significantly less in the 12 mmHg when compared with those in the 15 mmHg group^22^. In addition, one RCT compared surgical outcomes and postoperative complications between patients undergoing RARP at pneumoperitoneum pressure of 12 mmHg and 8 mmHg^23^. They found that postoperative ileus rates were significantly lower with no significant differences in estimated blood loss and operative time in the 8 mmHg group^23^. In the present study, all RARP procedures were conducted at the pneumoperitoneum pressure of 10 mmHg and PMI was associated with surgical outcomes. PMI may have stronger clinical significance in a much lower pneumoperitoneum such as 8 mmHg since working space may be less in such condition. However, future study is required to confirm this theory.

The present study showed that PV showed significant association with blood loss and longer console time. Interestingly, PMI and PV-to-PCI had higher risk than PV. This indicates that the size with regards to both the structural contents of the pelvic space and the pelvis itself is responsible for the surgical performance. Indeed, PMI is a parameter composed of both elements, since the value is a subtraction of the width of the prostate from the width of the inner pelvis. On top of this, the port is usually placed in a row near the level of umbilicus from left to right. Mechanically, the maneuver of the surgical robot may be more difficult in a pelvic cavity with shorter width, since the robotic arms have smaller space to mobilize or may have interference with one another. This is easily acknowledged in the Retzius-sparing RARP procedure, where the surgeon places the ports medially than conventional RARP, usually with an angle to proceed in a small space around the prostate, especially in the dorsal zone of the prostate and near the prostatic apex.

In the present study, the median difference of blood loss and console time in low PMI group (≤ 5.0 mm) and high PMI group (> 5.0 mm) were 400 mL and 30 min, respectively. Previous reports have indicated that operative time has significant impact on complications after surgery including surgical site infections (SSI), deep venous thrombosis (DVT)/pulmonary embolism (PE), and positioning injuries^24–26^. A study investigating SSI rates in 33 hospitals showed that operative time was 25 min longer in hospitals that had high outliers for SSI^24^. Another study by Abel et al. showed that an increase of 30 or 60 min was associated with 1.6 or 2.8 times increased risk of VTE^25^. In a study investigating positioning injuries after robotic assisted urological surgeries, 6.6% had positioning injuries and was significantly associated with operative time, although specific values of risks were not documented^26^. Not only does operating time impact clinical outcomes but also influence cost benefits. According to a report by Macario A et al., one minute of operating room time is calculated to result in an excessive cost of approximately 15 US dollars, which may add up to a significant amount of money in an annual based calculation^27^. Consequently, PMI may provide beneficial preoperative information to both surgeons and patients, since it is an indicator of less console time.

Our study has several limitations. First, the present study was conducted in a Japanese male population of which the prostate size was relatively smaller than those of Western countries^28,29^. However, the median preoperative prostate size was 28.5cm3 which was in line with the results of other Japanese cohorts^30–32^. Second, PMI may be useful in surgical approaches such as retroperitoneal approach or Retzius-sparing procedure since these approaches are known to have less working space^33^. However, in the present study all patients underwent RARP in a transperitoneal approach and one cannot judge the effectiveness of this tool in other approaches. Third, in the present study, the data on the source of bleeding was unavailable which would have given more insights to the correlation between PMI and the amount of bleeding regarding specific surgical procedures.

In conclusion, PMI can predict the surgical difficulty of the RARP procedure preoperatively and also may provide useful information to patients. Prospective and randomized studies are necessary to confirm our present study.

## Supplementary Information

Supplementary Information.

## Data Availability

The datasets used for the current study are not publicly available since ongoing clinical studies based on the same database are on progress, but it can be used by a reasonable request to the corresponding author.
