# Supplementary material for: The ‘prostate-muscle index’: a simple pelvic cavity measurement predicting estimated blood loss and console time in robot-assisted radical prostatectomy
Source: Sci Rep. 2022 Jul 13;12:11945. doi: 10.1038/s41598-022-16202-6 (PMC9279306; doi:10.1038/s41598-022-16202-6)
Supplement: Supplementary file 1 — Supplementary Information. [file 41598_2022_16202_MOESM1_ESM.docx]

Supplementary Table S1 Patient characteristics and surgical data of the entire cohort including cases with RARP with/without pelvic lymphadenectomy (N = 649)

| Variable | Value |
| --- | --- |
| Age, years, median (IQR) | 68 (64-72) |
| BMI, kg/m^2^, median (IQR) | 23.9 (21.9-25.6) |
| PSA, ng/mL, median (IQR) | 7.6 (5.6-11.3) |
| Preoperative PV, cm^3^, median (IQR) | 28.6 (22.8-37.7) |
| Prostate width, mm, median (IQR) | 48.0 (44.4-52.0) |
| Prostate height, mm, median (IQR) | 35.9 (32.2-40.3) |
| Prostate length, mm, median (IQR) | 32.6 (29.1-36.8) |
| Pathological T stage, N (%) |  |
| T2 | 445 (68.7) |
| T3 | 199 (30.7) |
| T4 | 4 (0.6) |
| Gleason score (GS), N (%) |  |
| < 8 | 480 (74.0) |
| ≥ 8 | 169 (26.0) |
| Surgery time, min, median (IQR) | 225 (178-264) |
| Console time, min, median (IQR) | 168 (129-208) |
| Blood loss, ml, median (IQR) | 300 (100-500) |
| Nerve-sparing |  |
| No | 478 (73.7) |
| Unilateral | 161 (24.8) |
| Bilateral | 10 (1.5) |
| Positive surgical margin, N (%) | 148 (22.8) |
| pT2 | 57 (8.8) |
| pT3 | 87 (13.4) |
| pT4 | 4 (0.6) |
| Blood transfusion, N (%) | 30 (4.6) |
| Lymph node dissection |  |
| No | 511 (78.8) |
| Limited | 90 (13.8) |
| Extended | 48 (7.4) |

IQR: interquartile range, BMI: body mass index, PSA: prostate-specific antigen, PV: prostate volume

Supplementary Table S2 Pelvimetric dimensions of the entire cohort including cases with RARP with/without pelvic lymphadenectomy (N = 649)

| Variable | Value |
| --- | --- |
| Median ASP, degrees, (IQR) | 72.8 (68.8-77.0) |
| Median APO, mm, (IQR) | 81.3 (75.4-86.8) |
| Median PMI, mm, (IQR) | 7.9 (4.4-11.6) |
| Median API, mm, (IQR) | 115.2 (108.8-121.8) |
| Median PD, mm, (IQR) | 131.6 (125.4-138.6) |
| Median ISD, mm, (IQR) | 90.1 (85.0-94.5) |
| Median PCI, (IQR) | 7.8 (7.3-8.3) |
| Median PV-to-PCI, (IQR) | 3.6 (2.8-4.8) |

ASP: angle of the intersection of the straight lines extending from the tuber ischiadicum to the symphysis pubis, APO: distance between the anteroposterior diameter of pelvic midplane between lower tip of the symphysis pubis and the coccyx representing the pelvic outlet, PMI: prostate muscle index, API: distance between the sacral promontory to the most superior aspect of the pubic symphysis, PD: distance between the cranio-caudal distance from the sacral promontory to the lower inner pubic symphysis, ISD: narrowest distances between tips of the ischial spines, PCI: pelvic cavity index, PV-to-PCI: prostate volume-to-pelvic cavity index, IQR: interquartile range

Supplementary Table S3 Univariate analyses of the entire cohort showing relationship between PMI and surgical margin or urinary continence after RARP with/without lymphadenectomy

|  | Positive surgical margin | | Urinary incontinence | |
| --- | --- | --- | --- | --- |
| Parameters | OR (95% CI) | P-value | OR (95% CI) | P-value |
| PMI (mm)  (PMI ≤ 5.0 versus > 5.0) | 0.90 (0.59-1.36) | 0.6 | 0.84 (0.59-1.21) | 0.4 |

Logistic regression models were used for univariate analyses. P-value of < 0.05 was considered to be statistically significant. OR: odds ratio, CI: confidence interval, PMI: prostate-muscle index

Supplementary Table S4

Univariate and multivariate analyses of factors associated with ‘blood loss > 250 mL’ in a subset of patients undergoing RARP without pelvic lymphadenectomy (N = 511)

|  | Univariate | | Multivariate | |
| --- | --- | --- | --- | --- |
| Parameters | OR (95% CI) | P-value | OR (95% CI) | P-value |
| BMI (kg/m^2^)  (BMI ≥ 24.0 versus < 24.0) | 2.56 (1.78-3.67) | **<** **0.0001** | 1.90 (1.23-2.93) | **0.003** |
| PV (cm^3^)  (PV ≥ 30.0 versus < 30.0) | 2.25 (1.57-3.23) | **<** **0.0001** |  |  |
| PV-to-PCI  (PV-to-PCI ≥ 4.0 versus < 4.0) | 3.06 (2.07-4.52) | **< 0.0001** | 1.77 (1.13-2.78) | **0.01** |
| PMI (mm)  (PMI ≤ 5.0 versus > 5.0) | 11.4 (6.73-19.4) | **<** **0.0001** | 9.96 (5.45-18.2) | **<** **0.0001** |
| Surgical experience  (Volume ≤ 25 versus > 25) | 1.72 (1.21-2.44) | **0.002** | 1.37 (0.89-2.12) | 0.1 |
| ASP (degree)  (ASP < 73.0 versus ≥ 73.0) | 1.39 (0.98-1.97) | 0.06 |  |  |
| APO (mm)  (APO ‹ 81.0 versus ≥ 81.0) | 0.73 (0.50-1.07) | 0.1 |  |  |
| Prostate width (mm)  (> 50.0 vs ≤ 50.0) | 1.54 (1.07-2.22) | **0.02** |  |  |
| Prostate length (mm)  (≥ 30.0 vs < 30.0) | 1.39 (0.93-2.03) | 0.1 |  |  |
| Prostate height (mm)  (≥ 35.0 vs < 35.0) | 2.04 (1.42-2.93) | **0.001** |  |  |

Logistic regression models were used for univariate and multivariate analyses. P-value of < 0.05 was considered to be statistically significant. OR: odds ratio, CI: confidence interval, BMI: body mass index, PV: prostate volume, PCI: pelvic cavity index, PMI: prostate-muscle index, ASP: angle of the symphysis pubis, APO: anteroposterior diameter of the pelvic outlet

Supplementary Table S5

Univariate and multivariate analyses of factors associated with ‘console time ≥ 160 minutes’ in a subset of patients undergoing RARP without pelvic lymphadenectomy (N = 511)

|  | Univariate | | Multivariate | |
| --- | --- | --- | --- | --- |
| Parameters | OR (95% CI) | P-value | OR (95% CI) | P-value |
| BMI (kg/m^2^)  (BMI ≥ 24.0 versus < 24.0) | 1.61 (1.13-2.30) | **0.009** | 1.63 (1.00-2.67) | **0.04** |
| PV (cm^3^)  (PV ≥ 30.0 versus < 30.0) | 1.52 (1.07-2.18) | **0.02** |  |  |
| PV-to-PCI  (PV-to-PCI ≥ 4.0 versus < 4.0) | 1.79 (1.22-2.62) | **0.003** | 1.43 (0.86-2.380) | 0.1 |
| PMI (mm)  (PMI ≤ 5.0 versus > 5.0) | 2.88 (1.90-4.35) | **<** **0.0001** | 2.28 (1.29-4.04) | **0.004** |
| Surgical experience  (Volume ≤ 25 versus > 25) | 14.8 (9.63-22.7) | **<** **0.0001** | 16.1 (9.92-26.1) | **< 0.0001** |
| ASP (degree)  (ASP < 73.0 versus ≥ 73.0) | 1.41 (0.99-2.00) | 0.051 |  |  |
| APO (mm)  (APO < 81.0 versus ≥ 81.0) | 0.85 (0.58-1.25) | 0.4 |  |  |
| Prostate width (mm)  (> 50.0 vs ≤ 50.0) | 1.56 (1.08-2.25) | **0.02** |  |  |
| Prostate length (mm)  (≥ 30.0 vs < 30.0) | 1.09 (0.74-1.61) | 0.6 |  |  |
| Prostate height (mm)  (≥ 35.0 vs < 35.0) | 1.50 (1.05-2.10) | **0.02** |  |  |

Logistic regression models were used for univariate and multivariate analyses. P-value of < 0.05 was considered to be statistically significant. OR: odds ratio, CI: confidence interval, BMI: body mass index, PV: prostate volume, PCI: pelvic cavity index, PMI: prostate-muscle index, ASP: angle of the symphysis pubis, APO: anteroposterior diameter of the pelvic outlet

Supplementary Table S6

Univariate and multivariate analyses of factors associated with ‘blood loss > 350 mL’ in patients undergoing RARP with pelvic lymphadenectomy (N = 138)

|  | Univariate | | Multivariate | |
| --- | --- | --- | --- | --- |
| Parameters | OR (95% CI) | P-value | OR (95% CI) | P-value |
| BMI (kg/m^2^)  (BMI ≥ 24.0 versus < 24.0) | 3.01 (1.49-6.09) | **0.003** | 2.14 (0.95-4.82) | 0.06 |
| PV (cm^3^)  (PV ≥ 30.0 versus < 30.0) | 2.47 (1.23-4.93) | **0.01** |  |  |
| PV-to-PCI  (PV-to-PCI ≥ 4.0 versus < 4.0) | 2.50 (1.21-5.17) | **0.01** | 2.17 (0.95-4.98) | 0.06 |
| PMI (mm)  (PMI ≤ 5.0 versus > 5.0) | 5.17 (2.28-11.7) | **< 0.0001** | 3.60 (1.43-9.00) | **0.006** |
| Surgical experience  (Volume ≤ 25 versus > 25) | 2.76 (1.38-5.52) | **0.004** | 2.69 (1.19-6.05) | **0.01** |
| ASP (degree)  (ASP < 73.0 versus ≥ 73.0) | 0.72 (0.36-1.41) | 0.3 |  |  |
| APO (mm)  (APO ‹ 81.0 versus ≥ 81.0) | 1.52 (0.84-3.50) | 0.1 |  |  |
| Prostate width (mm)  (> 50.0 vs ≤ 50.0) | 1.71 (0.48-2.57) | 0.8 |  |  |
| Prostate length (mm)  (≥ 30.0 vs < 30.0) | 2.55 (0.96-6.73) | 0.06 |  |  |
| Prostate height (mm)  (≥ 35.0 vs < 35.0) | 1.44 (0.73-2.86) | 0.3 |  |  |

Logistic regression models were used for univariate and multivariate analyses. P-value of < 0.05 was considered to be statistically significant. OR: odds ratio, CI: confidence interval, BMI: body mass index, PV: prostate volume, PCI: pelvic cavity index, PMI: prostate-muscle index, ASP: angle of the symphysis pubis, APO: anteroposterior diameter of the pelvic outlet

Supplementary Table S7

Univariate and multivariate analyses of factors associated with ‘console time > 200 minutes’ in a subset of patients undergoing RARP with pelvic lymphadenectomy (N = 138)

|  | Univariate | |
| --- | --- | --- |
| Parameters | OR (95% CI) | P-value |
| BMI (kg/m^2^)  (BMI ≥ 24.0 versus < 24.0) | 1.83 (0.92-3.65) | 0.08 |
| PV (cm^3^)  (PV ≥ 30.0 versus < 30.0) | 1.17 (0.59-2.30) | 0.7 |
| PV-to-PCI  (PV-to-PCI ≥ 4.0 versus < 4.0) | 1.43 (0.70-2.90) | 0.3 |
| PMI (mm)  (PMI ≤ 5.0 versus > 5.0) | 1.26 (0.61-2.60) | 0.5 |
| Surgical experience  (Volume ≤ 25 versus > 25) | 7.57 (3.52-16.2) | **<** **0.0001** |
| ASP (degree)  (ASP < 73.0 versus ≥ 73.0) | 1.50 (0.76-2.96) | 0.3 |
| APO (mm)  (APO < 81.0 versus ≥ 81.0) | 0.75 (0.36-1.56) | 0.4 |
| Prostate width (mm)  (> 50.0 vs ≤ 50.0) | 1.51 (0.74-3.10) | 0.2 |
| Prostate length (mm)  (≥ 30.0 vs < 30.0) | 0.91 (0.36-2.27) | 1.0 |
| Prostate height (mm)  (≥ 35.0 vs < 35.0) | 1.27 (0.64-2.51) | 0.6 |

Logistic regression models were used for univariate and multivariate analyses. P-value of < 0.05 was considered to be statistically significant. OR: odds ratio, CI: confidence interval, BMI: body mass index, PV: prostate volume, PCI: pelvic cavity index, PMI: prostate-muscle index, ASP: angle of the symphysis pubis, APO: anteroposterior diameter of the pelvic outlet
